# Improving Anemia Assessment in Clinical and Public Health Settings

**DOI:** 10.1016/j.tjnut.2023.05.032

**Published:** 2023-09-29

**Authors:** Anne M. Williams, Kenneth H. Brown, Lindsay H. Allen, Omar Dary, Denish Moorthy, Parminder S. Suchdev

**Affiliations:** 1 Department of Human Nutrition, University of Otago, Dunedin, New Zealand; 2 Nutrition Branch, Centers for Disease Control and Prevention, Atlanta, GA, United States; 3 Emory University, Atlanta, GA, United States; 4 Department of Nutrition and Institute for Global Nutrition, University of California, Davis, CA, United States; 5 United States Department of Agriculture, Agricultural Research Service Western Human Nutrition Research Center, Davis, CA, United States; 6 Division of Nutrition and Environmental Health, Office of Maternal and Child Health and Nutrition, Bureau for Global Health, United States Agency for International Development, Washington, DC, United States; 7 USAID Advancing Nutrition, Arlington, VA, United States

**Keywords:** hemoglobin, anemia assessment, anemia etiology, population-based methods

## Abstract

We aim to provide a practical approach to assess anemia and its primary causes, both in clinical settings and in the context of public health programs. Anemia remains a global challenge; thus, to achieve goals for anemia reduction and assess progress, standardized approaches are required for the assessment of anemia and its causes. We first provide a brief review of how to assess anemia, based on hemoglobin concentrations and cutoffs that correspond to age, sex, and physiologic status. Next, we discuss how to assess the likely causes of anemia in different settings. The causes of anemia are classified as non-nutritional (for example, because of infection, inflammation, blood loss, or genetic disorders) or nutrition-specific (for example, because of deficiencies of iron, vitamin A, riboflavin, vitamin B_12_, or folate). There is an important overlap between these 2 categories, such as the increased likelihood of iron deficiency in the context of inflammation. Given the multifaceted nature of anemia etiology, we introduce a framework for anemia assessment based on the “ecology of anemia,” which recognizes its many overlapping causes. This conceptual framework is meant to inform what data on anemia causes may need to be collected in population surveys. The framework has a supporting table with information on the diagnostic tests, biomarkers and proposed cutoffs, characteristics, and feasibility of collecting the myriad information that can help elucidate the anemia etiology. We also provide examples of how this framework can be applied to interpret the anemia risk factor data from population-based surveys that can inform decisions about context-specific interventions. Finally, we present research gaps and priorities related to anemia assessment.

## Introduction

Anemia, defined as low hemoglobin (Hb) concentration in the blood, remains a critical global public health problem that contributes to increased morbidity and mortality, particularly in women and preschool-age children [1,2]. Globally, regionally, and in nearly all countries, the progress on anemia reduction in women aged 15–49 y is insufficient to meet the World Health Assembly global nutrition target to halve anemia prevalence by 2025, and the prevalence of anemia in children also remains high [3].

A key challenge to quantifying the global burden of anemia is the variability of anemia prevalence estimates and trends resulting from the variety of different assessment techniques used to generate these data. For example, most of the anemia data in the 2022 paper by Stevens et al. [3] came from population surveys that utilized capillary blood samples, which can produce variable results of Hb concentration compared with venous blood [4-6]. Therefore, the high anemia prevalence may be partially because of measurement bias.

To make progress on global goals to reduce anemia and to determine the effectiveness of interventions, appropriate assessment tools to reliably measure and interpret Hb concentrations and determine the underlying causes of anemia in both clinical and community settings are necessary. Inadequate red blood cell (RBC) production, decreased RBC life span, increased blood loss, or combinations of these conditions result in anemia, which reduces the oxygen-carrying capacity of RBC. Anemia, like stunting, is a condition reflective of diverse and overlapping causes, that is, an ecology involving nutrition, genetics, disease, structural and social determinants, and the environment. Understanding the components of the ecology of anemia is critical for our efforts to identify and treat anemia with precision at both individual and population levels. Here we present a summary of current information on anemia assessment from the USAID (United States Agency for International Development) Advancing Nutrition Anemia Task Force.

### Important considerations for anemia assessment

Why is anemia assessment important—and why is measuring Hb [or hematocrit (Hct)] alone insufficient? First, anemia assessment identifies individuals and populations who may benefit from, or be harmed by, an intervention. These can be unique groups based on environmental (for example, malarial regions), genetic (for example, those with inherited blood disorders), or demographic (for example, pregnant women and young children) characteristics. Anemia also reflects health risks; for example, pregnant women with anemia have a higher risk of negative consequences of blood loss during delivery. Assessment is important to target those disproportionately affected by anemia and to monitor and evaluate anemia control programs. Although Hb and Hct reflect the status or perturbations in the hematological system, they are not sufficient to make a differential diagnosis that reflects the range of etiologies, as detailed in the paper on the biology of anemia elsewhere in this supplement [7]. Assessment of the causes of anemia involves collecting biomarkers, which are objective measures of normal or pathogenic processes, or responses to a therapeutic intervention (for example, indicators of micronutrient status or inflammatory proteins) [8,9].

Anemia assessment tools include Hb, Hct, and the myriad information that informs the underlying causes of anemia. They should be valid (accurate), precise (reproducible and limited variability), affordable, feasible, and acceptable [9]. Factors such as age, sex, pregnancy status, and trimester influence Hb and Hct biomarker cutoffs, whereas pre-analytic, and analytic factors influence the quality of measurement. These factors, in addition to genetics, lifestyle (for example, smoking, altitude, diet), and inflammation need to be considered when assessing anemia.

As previously mentioned, we are not on track to meet the World Health Assembly goal to reduce the prevalence of anemia in women of reproductive age by 50% by the year 2025 [3]. Estimation of anemia prevalence and its etiologies have been in many instances suboptimal, and prevention and treatment efforts have been weighted toward the provision of iron. Furthermore, appreciating the complex etiology of anemia and transferring that knowledge to anemia control programs has taken time. A deeper understanding of the ecology of anemia is needed to make better progress. The WHO is currently revising global guidelines for anemia diagnosis and control. WHO has specified that understanding the role of infectious and environmental factors in anemia etiology is an important research priority [1,10].

## Current guidelines targeting populations at risk for anemia

Two factors impeding action to assess and address anemia are *1*) the lack of universal guidance to screen young children and pregnant women for anemia or iron deficiency, and *2*) the limitations of using Hb alone in anemia screening programs because it does not allow for assessment of its causes. For example, for infants and young children, the American Academy of Pediatrics recommends universal screening for anemia by measuring Hb concentration at ~1 y of age [11], including exploration of anemia causes [11,12], whereas the Centers for Disease Control and Prevention recommends screening for iron deficiency anemia at 9–12 mo of age, again at 15–18 mo, and then yearly until 5 y of age [13]. Conversely, the United States Preventive Service Task Force concluded in 2015 that there was insufficient evidence to recommend screening for iron deficiency anemia in young children in the United States [14]. The WHO does not provide guidance on screening young children for anemia or iron deficiency. For pregnant women, the American College of Obstetricians and Gynecologists recommends universal screening for anemia and treating those with iron deficiency anemia with supplemental iron, in addition to iron-containing prenatal vitamins [15], whereas the WHO recommends daily iron and folic acid supplementation to prevent anemia during pregnancy, but lacks guidance on universal screening for anemia or iron deficiency [16]. Individual countries are expected to adapt the WHO guidance to their local context and develop clinical guidance on anemia screening policies, depending on available resources. Guidelines on screening for anemia could be improved if they included a sensitive and specific measure of iron status because it is one important cause of anemia and because iron deficiency can cause lifelong neurological consequences [17].

### Aims of manuscript

The aims of this paper are to *1*) propose best practices to assess anemia and its main causes, both in clinical and public health settings in resource-limited settings within low-income countries, and *2*) identify key research gaps and priorities related to anemia assessment. Each section includes considerations for individual screening in clinical settings, followed by guidance for surveys, surveillance, and program monitoring in populations.

## Approaches to Assess the Presence of Anemia

### Clinical history and physical examination

The assessment of anemia in an individual begins with careful history taking and a detailed physical examination. The patient’s medical history should include questions about the history of anemia symptoms such as easy fatigability or malaise, any bleeding (in particular, gastrointestinal or heavy menstrual bleeding), family history of genetic disorders, current medication use, living in or having traveled to malaria-endemic areas or areas affected by other anemia-causing infectious diseases, and diet history [18]. The examiner should also look for signs of pallor of the skin and mucous membranes, specifically in the conjunctiva of the eyes, nail beds, and palms [19]. Although the sensitivity (52%) and specificity (75%) of clinical assessment to diagnose anemia are poor [20], clinicians could use a physical exam to detect severe anemia and refer a patient to a higher-level facility for further evaluation and treatment where laboratory assessment of Hb or Hct is not possible [21].

### Blood-based assessment

The presence of anemia is most often defined as low Hb concentration in the blood, measured in whole blood, from the venous or capillary collection, either by automated hematological analyzers or spectrophotometry. Simple portable devices based on spectrometry such as the HemoCue© are also available for rapid point-of-care assessment. Several noninvasive approaches to anemia assessment are being evaluated (for example, smartphones or pulse co-oximeters) [22-25] but will not be discussed as the process of validation by other researchers is underway. A noninvasive technology developed by Massimo has received approval in the United States by the Food and Drug Administration. The caveats of venous or capillary blood collection for the determination of Hb are discussed in a later section, and more detailed information on blood collection and management for assessing Hb can be found in Module 3 of the Micronutrient Survey Manual and Toolkit [26].

**FIGURE 1.**
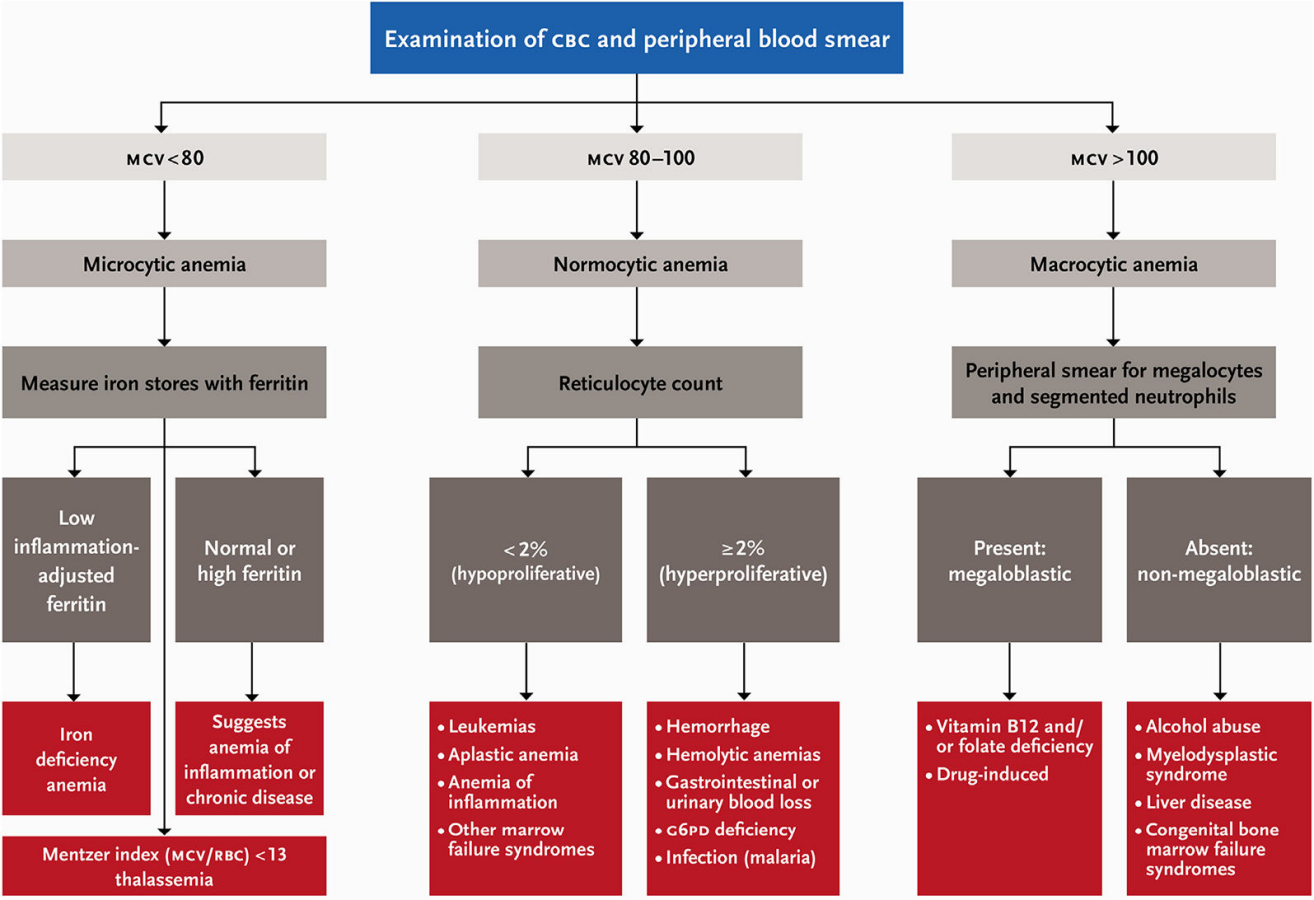
Clinical assessment of anemia etiology in individuals. Adapted from BMJ Best Practice 2019. CBC–complete blood count; G6PD–glucose-6-phosphate dehydrogenase; MCV–mean corpuscle volume; RBC–red blood cell count

The preferred method of Hb analysis is in the laboratory by automated hematology analyzers, which are advantageous because, in addition to Hb, they provide a complete blood count (CBC) including RBC indices, which can be useful for diagnosing the underlying cause of anemia (Figure 1). Other laboratory-based methods such as cyanmethemoglobin (Drabkin’s), Sahli’s, and the alkaline hematin method are rarely used, but may have utility in settings where automated instrumentation is not available [27].

Anemia can also be determined using Hct, which is the volume of RBCs relative to the volume of the blood sample, expressed as a percent. Automated hematology analyzers calculate Hct as the product of RBCs (millions/mm^3^) and the mean cell/corpuscular volume (in femtoliters) [28]. Hct does not supply additional information beyond that provided by the Hb concentration. Therefore, direct measurement of Hct is only suggested where Hb measurement is not feasible. Several factors affect the interpretation of Hb measurements, as described in the following sections. There has been consensus around the need to account for altitude and smoking status for >2 decades, which we describe first. That summary is followed by information summarizing the recent growing awareness of other sources of variability that need consideration when assessing Hb.

#### Adjusting Hb for altitude and smoking

The main function of Hb is to transport oxygen from the lungs to the tissues, and both altitude and cigarette smoking affect this vital function. Hb concentration increases in response to the decrease in partial pressure of oxygen at higher elevations. In a similar adaptation, Hb concentration increases among cigarette smokers because their Hb has a reduced oxygen-carrying capacity. WHO recommends adjusting measured Hb concentration for altitude and smoking status [29]. A recent report suggested that these adjustments be reexamined with newer data. The adjustments made as per 2001 WHO recommendations resulted in under-adjustments for light smokers and those residing at lower altitudes (that is, their adjusted Hb, when done, results in values higher than they should be), and over-adjustment for those residing at higher altitudes (their adjusted Hb values are lower than they should be) [30]. Because WHO is updating their recommended adjustment method in 2023, we advise practitioners to search for the most up-to-date guidance provided by WHO when they analyze Hb data. In the interim, the 2001 recommendations can be used.

#### Other sources of variability in Hb measurements

Venous or capillary blood samples can be used for Hb determination, and the method of blood collection can influence the Hb measurement [31-36]. Although the use of a single drop of capillary blood from a finger or heel stick is common in both clinical and public health settings, venous blood is preferable because of the large variation in Hb concentrations from the same individuals and human measurement error when using capillary blood [4-6]. A capillary blood sample can lead to erroneous results if there is a mixture of tissue fluid and skin, so phlebotomists should use standard practices that include quick, continuous, and deliberate skin puncture for a good flow of blood; wiping away the first drop of blood; and avoiding squeezing or milking the finger or heel [37]. A recent study in Mexico found as much as 20–30 g/L variation in capillary Hb estimates using HemoCue© compared with venous blood measured in a clinical hemocounter [38]. The Mexican study also found that a pool of capillary blood (several drops of blood collected by finger or heel pricks) may produce results similar to venous blood using HemoCue^®^. Further research to determine the appropriate methodologies for the collection, handling, and processing of pools of capillary blood is ongoing. Meanwhile, venous blood is preferred to have reliable results of Hb concentration unless it is confirmed that pools of capillary blood can produce reliable results.

The above-described results complement findings of other studies that have reported discrepancies in Hb concentration within individuals that are mainly due to factors such as the use of capillary (single drop and pooled) compared with venous blood [31,32,39-44] or the use of different HemoCue^®^ models [45]. Other preanalytical factors that can influence the measurement of Hb concentration with a HemoCue^®^ include *1*) deterioration of the microcuvette’s reagent because of humidity (especially in the HemoCue^®^ 201+ model), *2*) operational factors such as length of time lapse between loading the blood into the microcuvette and taking a reading with the equipment (HemoCue^®^ 301 model), and *3*) the care with which the cuvette is filled and loaded into the device [46,47].

#### Current cutoffs and challenges in interpreting Hb concentrations

Clinicians and other health practitioners diagnose anemia by applying different cutoffs to the measured concentration of Hb based on age, sex, and physiological status. WHO thresholds are widely applied to define anemia and its severity for various age and population groups [48]. The current cutoffs to define anemia in various age and population groups are as follows:

- children 6–59 mo: <110 g/L
- children 5–11 y: <115 g/L
- children 12–14 y: <120 g/L
- nonpregnant women of reproductive age (15–49 y): <120 g/L
- pregnant women: <110 g/L
- men (15 y and above): <130 g/L

A limitation of the current Hb thresholds that WHO uses to define anemia is that they are based on data from North America using statistical cutoffs (for example, the expectation that the lowest 5% of the population Hb distribution was abnormal, or representative of anemia) [13]. The need for separate cutoffs based on race has been proposed [49-52], as have revisions to the cutoffs for key age groups (for example, children younger than 24 mo of age) [53]. However, there is the concern that by applying different cutoffs for anemia by race, which is considered a social construct, it will exacerbate health inequities, and therefore it is not advisable [54]. Furthermore, it would not be feasible to systematically operationalize.

Sachdev et al. recently reported age and sex-specific Hb cutoffs to define anemia in children in India 1–19 y of age using data from the 2016 India Comprehensive National Nutrition Survey and applying a similar approach as current WHO Hb cutoffs using the lowest 5% of Hb distribution in a presumably healthy subpopulation of those sampled. This resulted in lower cutoffs than currently recommended by WHO [55]. Although these lower proposed Hb thresholds may be more appropriate for the Indian population, it is difficult to assess the validity of lower thresholds based solely on statistical cutoffs that have not been linked to physiologic or other health outcomes. Moreover, the use of country-specific cutoffs undermines the ability to compare data across countries.

Recent analyses from the International Fetal and Newborn Growth Consortium for the 21st Century and Biomarkers Reflecting Inflammation and Nutritional Determinants of Anemia (BRINDA) group also report Hb distributions where the 5th percentile was lower than the current WHO cutoffs to define anemia in apparently healthy pregnant women, children, and nonpregnant women of reproductive age [56,57]. Using data from 79,950 Hb observations from 30 population-based surveys covering all WHO geographic regions, the BRINDA team found that the WHO Hb cutoffs for defining anemia were higher than the 5th percentile of Hb distributions of all countries except the United States. This finding was held for children 6–59 mo and nonpregnant women 15–49 y and was reinforced using a physiologic biomarker of increased RBC production (soluble transferrin receptor). The Hb thresholds are currently under review by WHO based on the available data sources and the understanding that anemia would best be defined by clinical outcomes rather than population distributions [10].

In summary, the best practices for anemia assessment include the use of venous (or potentially pooled capillary) blood, Hb analysis by an automated analyzer or point-of-care HemoCue^®^ device using appropriate quality controls, Hb adjustment for altitude and smoking using updated WHO recommendations, and application of global age- and sex-specific and physiologically validated cutoffs for anemia. Application of the existing WHO Hb cutoffs is deemed to be the best alternative pending release of new recommendations.

## Approaches to Assess the Causes of Anemia

### Non-nutritional and nutrition-specific causes of anemia, and their interaction

Anemia will not be resolved unless its underlying causes are understood. In general, the causes of anemia can be categorized as non-nutritional or nutrition-specific and grouped by the mechanisms that produce anemia which include blood loss, increased hemolysis, decreased erythropoiesis, and micronutrient deficiencies (Table 1) [7]. Micronutrient deficiencies can be caused by insufficient dietary intake, impaired absorption, or increased losses. Factors that can influence nutrient absorption, such as celiac disease, loss of stomach acidification from advanced age, *Helicobacter pylori* infection or proton pump inhibitor or antacid use, are important to take into consideration when assessing anemia [7]. However, it is beyond the scope of this paper to detail all influencers of nutrient malabsorption.

It is important to recognize the reciprocal relationships among nutritional and non-nutritional causes of anemia as each cause may affect, and can be affected by, the other. For example, an individual may have acute inflammation because of an illness that could lead to functional iron deficiency, which is when the body may have sufficient iron, but it is sequestered because of elevated hepcidin from the inflammation. This individual would most likely be unable to absorb iron adequately from their diet or supplementation/fortification before the resolution of the inflammation. Similarly, overweight or obese individuals are at a greater risk for iron deficiency [58,59] because inflammation associated with obesity can reduce iron absorption and may increase iron sequestration [60,61]. In a recent trial, compared with infants born to normal weight women, infants born to overweight mothers had lower body iron stores because of decreased iron absorption late in pregnancy because of inflammation [62].

### Clinical assessment of anemia etiology

Clinical settings are convenient sites for determining the cause(s) of anemia in an individual, as the patient can be seen multiple times before and after initiating any treatments to alleviate the anemia. Once a clinician diagnoses a patient with anemia, most clinical algorithms start with evaluating the RBC indices from a CBC, which describe the size, shape, and Hb content of RBCs (Figure 1). For example, the mean corpuscular volume is the average size of the patient’s RBCs and can be low (microcytic), normal (normocytic), or high (macrocytic). The mean corpuscular hemoglobin is the average Hb content in RBCs and when low, can indicate iron deficiency or certain inherited blood disorders. Red cell distribution width (RDW) is a measure of the variation in RBC size, and a high RDW indicates a large variation in RBC sizes, as occurs with iron deficiency and with megaloblastic anemia. Nutritional causes of anemia can be ascertained using nutritional biomarkers, which are described in later sections.

Reticulocyte counts are also useful for establishing the general type of anemia. During the process of erythropoiesis, the bone marrow produces reticulocytes or immature RBCs. Under normal conditions, the rate of RBC production equals the rate of RBC loss, so reticulocytes make up ~1% of the circulating RBCs. However, after blood loss, with hemolytic anemias, or shortly after successful initiation of treatment for nutritional anemias, the reticulocyte count is elevated. Automated blood counters can count reticulocytes (Figure 1). Examination of a peripheral blood smear may also be helpful to assess RBC morphology and diagnose an inherited blood disorder, such as sickle cell anemia. Anemia because of underlying diseases, such as peptic ulcers, chronic kidney disease, uterine fibroids, or coeliac disease, may require additional workup and clinical referral to treat the root cause of the anemia. Figure 1 presents a proposed framework for anemia assessment in clinical settings.

### Population-based assessment of anemia etiology

We can use population-based surveys to estimate anemia prevalence and the strength of association between anemia and its causes. Decisions around which biomarkers to include in a survey depend on geographic locations (malaria compared with nonmalaria zone); available information on other underlying diseases (for example, selected parasites and genetic disorders); and financial and technical resources. The Micronutrient Survey Manual & Toolkit, produced by CDC, UNICEF, Nutrition International, and WHO, is a valuable resource covering multiple aspects of planning cross-sectional micronutrient surveys (available from https://mnsurvey.nutritionintl.org) [63].

Examples of population-based surveys and surveillance systems that collect Hb data include—

1. Demographic and Health Surveys (DHS) report on anemia prevalence in low- and middle-income countries and provide information on socio-demographic and geographic risk factors for anemia [64-67] and trend analyses [68,69].
2. The Guatemalan Epidemiological Health and Nutrition Surveillance System [70].
3. The Uganda National Panel Survey [71].
4. The Indian Comprehensive *National Nutrition Survey* [72].
5. The United States NHANES [73].
6. Malaria Indicator Surveys [74].
7. Population-based HIV Impact Assessment surveys [75-77].

**FIGURE 2.**
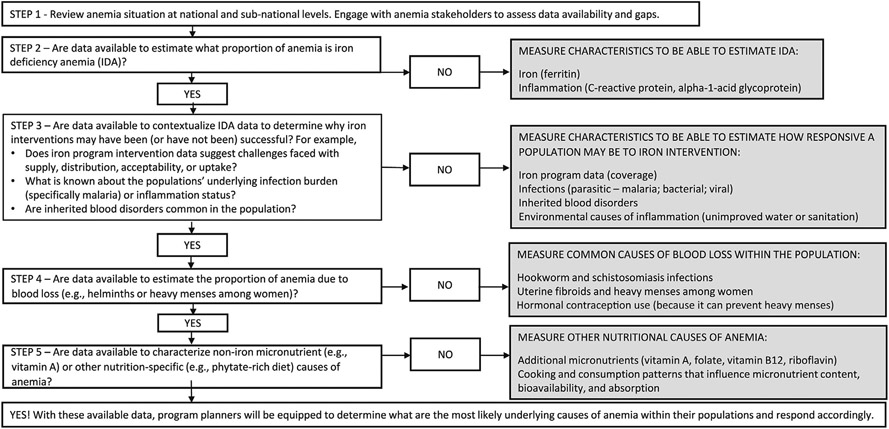
Conceptual framework to inform what information on the underlying causes of anemia to consider including in population-based surveys. Population assessment of the causes of anemia.

National nutrition surveys can collect individual-level information on the non-nutritional and nutritional causes of anemia, which provides the necessary data to model associations between anemia and its causes in populations [78-81]. One limitation of cross-sectional data interpretation is the inability to assign causal attribution. Intervention trials, cohort studies, or longitudinal population-based surveillance may be preferred for this purpose. However, to better understand the ecology of anemia within a population it may be prudent to look across multiple existing data sources to determine what the most likely underlying causes of anemia may be. This collation of data would also identify if future surveys could fill gaps in knowledge to better understand the ecology of anemia within a population. Figure 2 provides a conceptual framework that can be used to guide conversations and aid in decision making to determine what data to consider including in population-based surveys to better understand anemia ecologies within populations. This framework is meant to draw attention to the causes of anemia in addition to iron and the micronutrients that are commonly considered when discussing anemia. It is not a prescriptive one-size fits all formula.

In addition to these immediate causes of anemia, it is important to consider underlying structural and social determinants, such as economic stability; education access and quality; racism, discrimination, and violence; language and literacy skills; food security; and climate change and polluted environment [1,82].

Planning a population-based survey or setting up a health surveillance system that includes anemia and its underlying causes is a large undertaking. Several factors influence survey design, including the tools used for data collection and the specific biomarker(s) used to detect a particular condition. Sampling strategies, survey tools, and information to guide the selection of appropriate biomarkers of nutritional status are available online in the Micronutrient Survey Manual and Toolkit [63]. Table 2 provides a high-level overview of considerations for including items in a survey to assess anemia ecology, including feasibility of collection. Box 1 summarizes the anemia etiology components to consider in population surveys or surveillance.

#### Considerations for assessing status of iron and other relevant micronutrients

Although multiple indicators for iron status exist, WHO recommends assessing ferritin and inflammatory proteins to estimate the prevalence of iron deficiency in populations [83]. Additional micronutrient data, including vitamin A, vitamin B12, folate, and riboflavin, are useful for understanding the underlying causes of anemia in populations, as deficiencies in multiple micronutrients are common and can contribute to anemia. Dietary patterns that capture the intake of iron-rich food [84], iron inhibitors such as phytates and tannins, and other relevant micronutrients are also important to measure [85]. However, practitioners should not collect dietary data in place of nutritional biomarker data, but rather use it in conjunction with such data [86].

The list of causes of anemia to consider during survey planning may be sizable and will differ based on the infectious disease burden or environmental conditions of the setting. For example, groundwater iron may be important to assess [87,88]. If the infectious disease burden is high in a population, then a clear understanding of the disease interaction with the anemia ecology will be imperative, given the known downregulation of iron absorption and metabolic mobilization during bouts of infection or inflammation. Populations with lower infectious disease burden have been found to have a higher proportion of iron deficiency anemia [78,79,89], likely because infections are responsible for a larger proportion of anemia among populations with higher infectious disease burdens. We considered the BRINDA project and Global Burden of Disease (GBD) project, which estimated the global prevalence of anemia by cause [90, 91] when developing a decision-making framework to understand anemia causes at the population level (Figure 2).

In 2010, 4 of the 5 highest-ranking causes of anemia were non-nutritional: hookworm disease, sickle cell disorders, thalassemias, and malaria. Nevertheless, the GBD ranked iron deficiency as the leading cause of anemia, although the causal attribution model did not incorporate iron status data; researchers assigned it as the residual cause of anemia after identifying other causes [90]. Therefore, we emphasize the assessment of inherited blood disorders, infections, and iron status to understand the ecology of anemia (Figure 2).

## Utilizing survey data to inform programmatic decision making

Loechl et al. [92] in another paper (in this supplement) present a framework for how to use surveillance data to inform intervention choice. Here we present a brief discussion of the implications of how anemia survey data can inform programmatic decision making.

After the collection of Hb data alongside conditions known to cause anemia, it is useful to interpret the data and apply the results to inform public health anemia control programs. We use data from a national micronutrient survey in Malawi as an illustration of how to quantify the contributions of multiple anemias causes that often overlap with one another. Data from this survey were selected because they are unique in 2 ways: *1*) the data are publicly available and were collected in tandem (that is, linkable) with the DHS and *2*) the survey collected a wide variety of risk factors for anemia (for example, inherited blood disorders, infectious diseases, and multiple micronutrient deficiencies). Examining prevalence estimates of conditions known to cause anemia is a first step in understanding the ecology of the anemia. A common next step is to examine the univariate associations between risk factors and anemia (the outcome), and then build multivariable models predicting likely causes of anemia controlling for the overlap and interrelation between these conditions. The prevalence of conditions known to cause anemia, and the estimates of anemia among those with or without the conditions are presented in Table 3 using publicly available data from the 2015–2016 national micronutrient survey in Malawi. In unadjusted weighted univariate analyses, there was a significant association between any inflammation and anemia (prevalence ratio of 1.6, Table 3), but after controlling for iron deficiency, malaria and other covariates, the association between anemia and inflammation was attenuated (adjusted prevalence ratio of 1.2) given the overlapping of iron deficiency, malaria, and inflammation. The adjusted prevalence ratios indicate a stronger association between anemia and malaria than iron deficiency (Table 3). Similarly, in a cohort of children in Zambia, researchers attributed more anemia to malaria than iron deficiency in the high malaria season [93].

A national survey in Azerbaijan similarly used Poisson regression to calculate risk ratios and estimated that 17.6% of anemia was attributable to iron deficiency among children [94]. Therefore, Azerbaijan may potentially reduce ≤17.6% of the anemia burden if officials could eliminate iron deficiency among children. A recent comparative analysis of methods for estimating attributable fractions concluded that Poisson regression was the most accessible method for quantifying attribution using cross-sectional data [95]. Accounting for confounding and correlation among exposure variables is important to prevent overestimation of attribution of risk factors in regression models; therefore, measurement of multiple underlying risk factors for anemia within surveys is useful.

**FIGURE 3.**
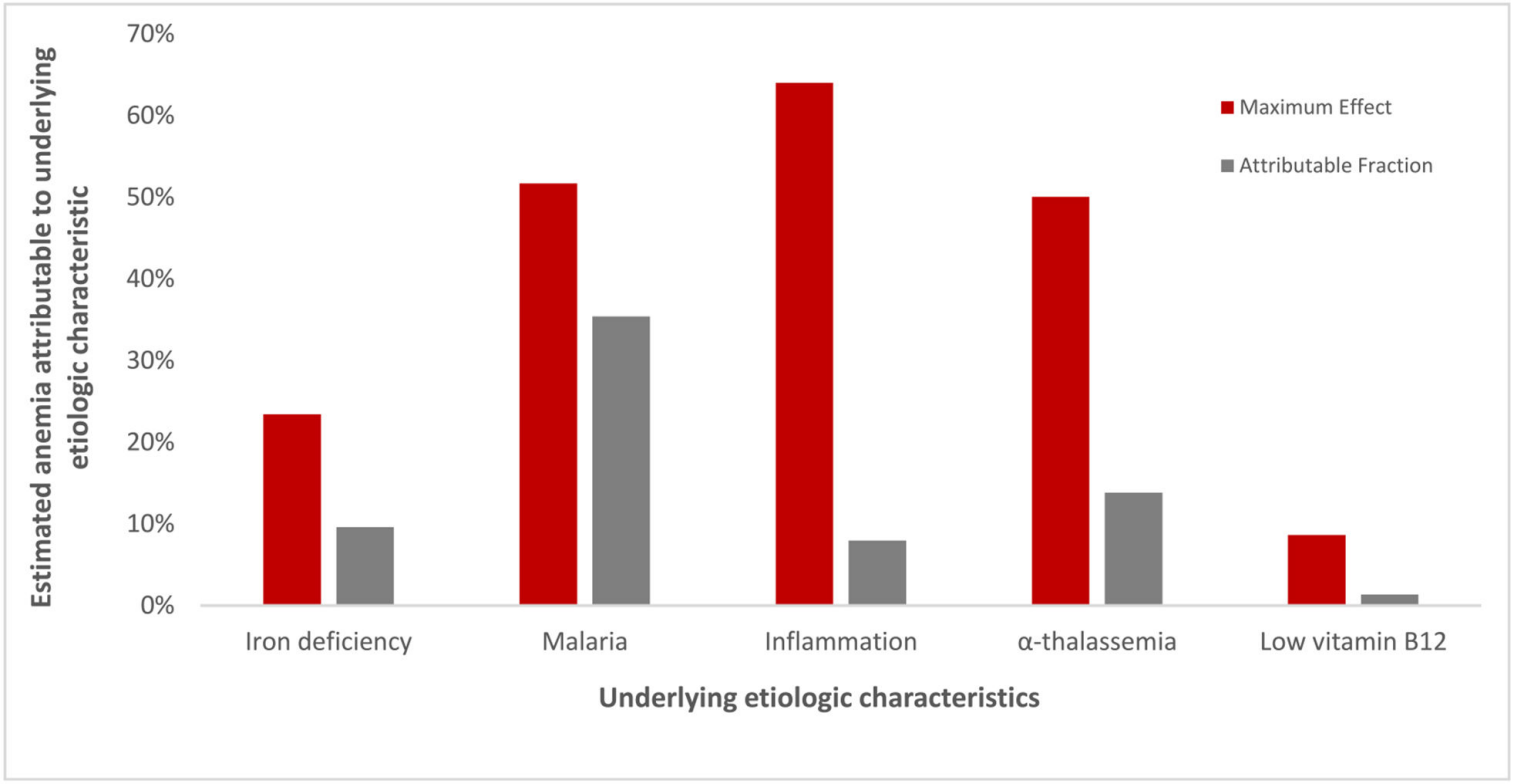
An illustrative example of overestimation of attribution to anemia when not accounting for multiple underlying characteristics: national survey data children 6–59 mo, Malawi 2015–2016 (*n* = 819). The maximum effect is an illustrative overestimation of how much anemia might be removed if the condition was ameliorated from the population. The maximum effect was calculated by dividing the prevalence of anemia and the condition (for example, iron deficiency anemia) by the prevalence of anemia. The attributable fraction was calculated as the proportion exposed * (RR − 1) / [1 + proportion exposed * (RR − 1)], where the adjusted prevalence ratio was substituted for the RR. Iron deficiency was defined as inflammation-adjusted serum ferritin using the Biomarkers Reflecting Inflammation and Nutritional Determinants of Anemia method; inflammation was defined as CRP >5 mg/L or AGP >1 g/L; alpha-thalassemia is the presence of 1 or 2 deletions; and low vitamin B12 was defined as <220 pmol/L. AGP, alpha-1-acid glycoprotein; CRP, C-reactive protein; RR, relative risk.

It is known that attributable fractions likely overestimate the benefit of iron interventions on anemia in settings where multiple causes of anemia overlap [96]. Furthermore, estimating attributable fractions from cross-sectional data is unconventional because you cannot calculate risk without a time component, and the attributable fraction percentages can total to over 100%. We show an illustrative example of the overestimation of attributable fractions using data from Malawi without accounting for the overlap of conditions (for example, malaria + iron deficiency + inflammation) within individuals. We calculated the maximum expected effect by dividing the prevalence of anemia and the condition (for example, iron deficiency) by the prevalence of anemia. The attributable fraction was calculated as the proportion exposed * (RR − 1) / [1 + proportion exposed * (RR − 1)], where the adjusted prevalence ratio is substituted for the relative risk (RR). This simplistic approach overestimates the effect that removing single conditions would have on the anemia prevalence. For example, among complete cases with no missing data for covariates, dividing the prevalence of children with anemia and malaria (15%) by the prevalence of children with anemia (29%) results in 51.5%, which suggests that eliminating malaria among children could reduce more than half of child anemia. However, we know that some children concurrently have malaria, iron deficiency, and inflammation (as well as inherited blood disorders or other micronutrient deficiencies). If we use the adjusted prevalence ratio representing the influence of malaria on anemia (3.1) to estimate RR in an attributable fraction equation, then the percent of anemia amenable to the elimination of malaria among children would decrease to 35% (Figure 3). Although these analytic methods are imperfect, they inform the relative contribution of population characteristics to the ecology of anemia and highlight the importance of malaria elimination, for example, to achieve progress in anemia reduction.

## Evidence Gaps

We summarize the research needs for diagnosing anemia and determining its causes in Table 4. Key questions are categorized as follows: *1*) assessing Hb and anemia etiologies and *2*) population anemia etiology assessment. Essential to both sets of questions is the feasibility and cost-effectiveness of assessment tools. As researchers develop new methods for measuring Hb and novel biomarkers of anemia etiology, they will need to be assessed for both their sensitivity and specificity with respect to existing methods or markers as well as their suitability for population assessment. Criteria for assessing their utility include—

- relative ease of specimen collection, processing, and analysis;
- accuracy and precision of the test results;
- cost of the instrument or the laboratory analysis;
- acceptability of the method for the individuals being assessed [1].

Priority research questions on anemia assessment tools include procedures and methodologies to improve the reliability of results with point-of-care and noninvasive Hb instruments (for example, assessing venous compared with pools of capillary blood, validating the calibration and comparability use of devices in different settings, and developing approaches to account for preanalytical factors when possible). Development of point-of-care assessment tools that can simultaneously measure Hb as well as key anemia risk factors (for example, inflammation, iron deficiency, malaria, and hemoglobinopathies) is needed.

There are also important research questions related to population-based assessment of the ecology of anemia. For example, is it feasible logistically and would it be more cost-efficient to do a CBC and reticulocyte count on all survey participants and assess underlying causes of macrocytic or microcytic anemia purposefully rather than measure all possible causes of all forms of anemia in everyone? This could reduce the number of laboratory analyses (for example, assessing for iron status only among those with microcytosis or causes of hemolytic anemias only in those with elevated reticulocyte count), but may increase complexity, and the cost of that trade-off is unknown. This selective measurement of biomarkers may, however, have the unintended consequence of overlooking public health problems (for example, high prevalence of micronutrient deficiency) among segments of the population that do not have anemia. Finally, how to best measure underlying structural and social determinants of health and assess their impact on anemia burden remains a research gap.

In conclusion, the persistence of anemia within populations increasingly perplexes public health professionals. Our goal is to encourage the assessment of non-nutritional causes of anemia, as well as the role of nutrients in addition to iron, to better understand the ecology of anemia (Box 2). Population-based surveys that take an ecological approach to collecting data on infections such as malaria and hookworm, inflammation, inherited blood disorders, and iron status offer an opportunity to assess the relative contributions of non-nutritional and nutritional etiologies of anemia. Such survey results can be used to prioritize interventions that would ostensibly reduce the anemia burden.

## Figures and Tables

**TABLE 1:** Common non-nutritional and nutritional causes of anemia^1^

Blood loss	Non-nutritional causes of anemia			Nutrition-specific causes of anemia	
	Increased hemolysis	Deficient erythropoiesis		Microcytic	Macrocytic
		Microcytic	Normocytic		
Heavy menstrual bleeding Postpartum hemorrhage Gastrointestinal blood loss (hookworm infection, ulcers, and schistosomiasis) Urinary blood loss (schistosomiasis)	Infection (malaria) Hemoglobin disorders (sickle cell disease, and thalassemia) Enzymopathies (glucose 6 phosphate dehydrogenase [G6PD] deficiency) Autoimmune hemolytic anemia Intravascular destruction of RBC (transfusion reactions, hemolytic uremic syndrome, and snake bites) Hypersplenism	Anemia of inflammation (chronic disease) Heterozygous thalassemia trait Homozygous/compound heterozygous thalassemia	Anemia of inflammation (chronic disease) Chronic kidney disease Bone marrow failure (aplastic anemia and leukemia)	Iron deficiency Vitamin A deficiency Riboflavin deficiency (normocytic)	Folate deficiency Vitamin B12 deficiency

Abbreviation: RBC, red blood cell.

1 Not all potential causes of anemia are identified in this list. Nutrient-specific causes can be caused by insufficient dietary intake, impaired absorption, or increased losses. We discuss the causes that are more prevalent at the population-level in resource-limited settings within low-income countries that are modifiable by public health interventions (discussed by Loechl et al. [92]). This table is adapted from Chaparro and Suchdev [1].

**TABLE 2:** Considerations to assess selected causes of anemia in surveys, surveillance, or program monitoring

Cause of anemia	Diagnostic test, biomarker, or characteristics to identify condition	Proposed cutoff values or defining characteristics	Feasibility of collection
Iron deficiency	Ferritin CRP and AGP	Infants and children <5 y (<12 μg/L); children, adolescents, and adults 5 y and older (<15 μg/L); and pregnant women (<15 μg/L) Inflammation defined as CRP >5 mg/L or AGP >1 g/L	Often requires venous blood collection but a sandwich ELISA technique enables collection of pools of capillary blood; cold chain required. Biomarkers of inflammation are required for interpretation of ferritin
Infections	Malaria: blood film, RDT, and PCR Hookworm: stool examination (Kate Katz, fixed sample, and PCR) Schistosomiasis: microscopy/Ag Viral (HIV/acquired immunodeficiency syndrome, hepatitis C, and respiratory viruses including COVID-19) Tuberculosis: Sputum AFBs, PCR, and Mantoux/quantiferon for latent Salmonella, *H. pylori*, and other bacterial infections	Usually defined by presence of infectious organism	Malaria and hematuria can be measured in the field from capillary blood and urine, respectively; PCR testing diagnostic capacity variable for other infections
Inflammation	CRP and AGP	Inflammation defined as CRP >5 mg/L or AGP > 1 g/L	Blood collection (venous or pooled capillary); cold chain required
Inherited blood disorders	Abnormalities of Hb synthesis (alpha or beta thalassemia) Abnormalities of Hb structure (Hb S, C, and E) Abnormalities of red cell enzymes (G6PD deficiency) Abnormalities of RBC membrane (hereditary spherocytosis and elliptocytosis)	Usually defined with genetic test using deoxyribonucleic acid extracted from whole blood in tube or extracted from dried blood cards Hb electrophoresis can also be used to detect Hb S, C, and thalassemia	May be collected using dried blood cards, so is not dependent on venous blood collection or cold chain
Blood loss	Hormonal contraceptive use (beacuse it can prevent heavy menses) Heavy menses Uterine fibroids	Usually defined by participant recall to questionnaires that include information about these characteristics	Requires knowledge of cultural norms around reproductive health and birth
Additional micronutrients	Vitamin A Riboflavin Folate Vitamin B_12_	Serum/plasma retinol <0.7μmol/L (although for women is still uncertain) Erythrocyte gluthathione reductase activity coefficient >1.3 Serum folate <6.8 nmol/L (risk of megaloblastic anemia) or RBC folate <100 ng/mL assayed by *Lactobacillus casei* Vitamin B12 <150 pmol/L (risk of megaloblastic anemia)	Requires venous blood collection and cold chain; limited availability of laboratories with externally validated performance
Lack of micronutrients and diversity in diet	Usual intake of animal source foods or fortified foods Usual intake of iron inhibitors (phytates and tannins) Cooking and consumption practices that influence micronutrient content, bioavailability, and absorption	Usually defined by participant recall to questionnaires that include information about these characteristics	Requires knowledge of common dietary and cooking patterns among target population

Abbreviations: AFB, acid-fast bacillus; AGP, alpha-1-acid glycoprotein; CRP, C-reactive protein; G6PD, glucose-6-phosphate dehydrogenase; Hb, hemoglobin; PCR, polymerase chain reaction; RBC, red blood cell; RDT, rapid diagnostic test.

**TABLE 3:** Anemia was more strongly associated with malaria than iron deficiency among children 6–59 mo old, Malawi Micronutrient Survey 2015–2016 (*n* = 819)

Characteristics known to cause anemia (prevalence)	Anemia prevalence among exposed^1^	Anemia prevalence among unexposed^1^	Prevalence ratio	Adjusted prevalence ratio^2^
Iron deficiency^3^ (16.9%)	40.2	26.9	1.5	1.6
Malaria (26.2%)	57.4	19.1	3.0	3.1
Inflammation^4^ (53.0%)	35.2	22.3	1.6	1.2
Alpha-thalassemia (42.7%)	34.1	25.4	1.3	1.4
Low vitamin B_12_ (5.3%)	47.0	28.1	1.7	1.3

1 Exposed vs. unexposed means those with the characteristic vs. those without the characteristics. For example, the anemia prevalence among children with iron deficiency was 40.2%, and the anemia prevalence among children without iron deficiency was 26.9%.

2 Adjusted prevalence ratio is the prevalence of anemia among exposed divided by the prevalence of anemia among unexposed, while accounting for other conditions significantly associated with anemia (iron, malaria, inflammation, alpha-thalassemia, vitamin B_12_ depletion, and child age).

3 Iron deficiency is defined as inflammation-adjusted ferritin <12 μg/L.

4 Inflammation is defined as C-reactive protein >5 mg/L or alpha-1-acid glycoprotein >1 g/L.

**TABLE 4:** Evidence gaps, by category of research

Assessing Hb and anemia etiologies	Population anemia etiology assessment
Noninvasive and other innovative methods to measure Hb Point-of-care tools that can simultaneously measure Hb as well as common anemia etiologies Point-of-care tools of iron status and metabolism (for example, ferritin, hepcidin, and erythropoietin) Approaches to verify Hb assessment performance and account for preanalytical factors that influence Hb concentrations Approaches to assess the role of multiple micronutrient deficiencies in anemia in different settings Cost-effective approaches to assessing inherited blood disorders, possibly based on initial screening of red blood cell indices Re-evaluation of Hb thresholds to define anemia	Statistical approaches such as population attributable risk to assess anemia etiologies using cross-sectional data Cost and logistical implications of broad-based anemia etiology assessment vs. stepwise diagnostic algorithm Best practices for assessing social and structural determinants of anemia

Hb, hemoglobin.

## BOX 1 Assessment Considerations for Anemia Etiology Surveillance or Population Surveys

If most anemia is NOT iron deficiency anemia, it will be more important to assess other causes of anemia (e.g., infections, inherited blood disorders, and blood loss) than iron alone. However, to determine how much of the anemia is IDA, an iron biomarker will need to be measured with Hb.

- Infections (e.g., important parasitic, viral, and bacterial disease burdens of the target population) especially malaria in malaria-endemic regions.
- Inflammation (C-reactive protein, alpha-1-acid glycoprotein) for its independent role in *anemia*.
- Inherited blood disorders
  - Alpha-thalassemia, which can cause mild anemia (Gahutu et al. 2012; McGann et al. 2018).
  - Other inherited blood disorders, including sickle cell hemoglobin and G6PD deficiency, may mitigate malarial infection and therefore influence anemia (Mockenhaupt et al. 2004; Uyoga et al. 2015).
- Blood loss: In settings where the underlying prevalence of blood disorders is unknown it is important to assess blood loss, by asking women of reproductive age about—
  - heavy menses, uterine fibroids
  - use of hormonal contraceptives that would reduce blood loss during the menstrual cycle, could also inform intervention strategies to mitigate anemia.
- Nutritional status including—
  - dietary intake
  - biomarkers of specific nutrients including iron and other relevant micronutrients.

## BOX 2 Key Take-Home Messages

- Anemia is itself not a disease, but a condition with diverse and overlapping causes.
- Broadening the focus from predominately iron to a better understanding of nutritional and non-nutritional anemia determinants would inform clinical care and anemia control programs.
- Adopting a practical, systematic approach to anemia assessment is essential to address data gaps and make progress on reducing the global burden of anemia.

## Abbreviations:

BRINDA: Biomarkers Reflecting Inflammation and Nutritional Determinants of Anemia
CBC: complete blood count
DHS: Demographic and Health Surveys
GBD: Global Burden of Disease
Hct: Hematocrit
Hb: hemoglobin
RDW: red cell distribution width
RBC: red blood cell
RR: relative risk
